# Impact of Sodium-Glucose Cotransporter-2 Inhibitors (SGLT2i) on Cardiovascular Outcomes in Heart Failure With Reduced Ejection Fraction Under Contemporary Standard Care: A Systematic Review and Meta-Analysis

**DOI:** 10.7759/cureus.99014

**Published:** 2025-12-11

**Authors:** Fahd Mohammed Abdullah Makhsham, Khaled Abdulbaqi Baggash Nasr, Amer Abdulelah Saif Al-Sewaiee, Hussam Salmen Abdulrahman Abdullah, Jiab Mahyoub Abdo Noman, Abdikader Abdullahi Salad, Ahmed Abdulwasea Furas Ali Mohsen, Abdullah Mohsan Nasser Saleh, Qingchun Zeng

**Affiliations:** 1 Guangdong Provincial Key Laboratory of Cardiac Function and Microcirculation, Cardiology Department, Nanfang Hospital, Southern Medical University, Guangzhou, CHN; 2 State Key Laboratory of Multi-Organ Injury Prevention and Treatment, Department of Cardiology, Nanfang Hospital, Southern Medical University, Guangzhou, CHN; 3 Guangdong Provincial Key Laboratory of Cardiac Function and Microcirculation, Cardiology Department, Guangdong Provincial People's Hospital, Guangdong Cardiovascular Institute, Southern Medical University, Guangzhou, CHN; 4 School of Pharmaceutical Sciences, Guangdong Provincial Key Laboratory of New Drug Screening, Southern Medical University, Guangzhou, CHN

**Keywords:** cardiovascular mortality, dapagliflozin, heart failure, sglt2 inhibitors, type 2 diabetes

## Abstract

This systematic literature review and meta-analysis evaluates the efficacy of sodium-glucose cotransporter-2 inhibitors (SGLT2i) in reducing key cardiovascular outcomes, namely hospitalization for heart failure, cardiovascular mortality, and all-cause mortality in patients with heart failure with reduced ejection fraction (HFrEF). A total of 12 publications (including subgroup analyses) were analyzed. The selected RCTs compared the effects of SGLT2i (empagliflozin, dapagliflozin, and canagliflozin) with standard care among patients with HFrEF. The pooled data demonstrated that SGLT2i significantly reduced the risk of cardiovascular mortality by 23% and hospitalization for heart failure by 25%, compared to standard care. These benefits were consistently observed across both diabetic and non-diabetic subgroups, underscoring the broader therapeutic potential of SGLT2i in HFrEF management. However, no statistically significant reduction in all-cause mortality was identified. While the findings affirm the cardioprotective effects of SGLT2i, the lack of impact on overall mortality highlights the need for further longitudinal studies. These results support the integration of SGLT2i into contemporary HFrEF treatment protocols while informing future research priorities.

## Introduction and background

Heart failure with reduced ejection fraction (HFrEF) is a widespread condition that declines the quality of life, increases morbidity and mortality rates, and leads to frequent hospitalizations [1]. This outcome may be due to injury to the cardiac muscles, resulting in a left ventricular ejection fraction (LVEF) of 40% or lower [2]. It is one of the main global causes of cardiovascular morbidity and mortality, currently affecting approximately 64.3 million individuals [3]. Common risk factors for heart failure include hypertension, diabetes, and less commonly discussed contributors such as HIV infection and social determinants of health, all of which can significantly influence outcomes [3]. Despite advances, patients with HFrEF continue to face significantly increased risks of disease progression, recurrent hospitalizations due to heart failure exacerbations, and cardiovascular mortality, even with newer pharmacologic therapies [4].

The goal of HFrEF management is to reduce symptoms, minimize hospitalizations, and prolong life via pharmacologic and non-pharmacologic strategies [5-7]. Guideline-directed medical therapy, including mineralocorticoid receptor antagonists, beta‑blockers, angiotensin receptor blockers (ARBs), and angiotensin‑converting enzyme (ACE) inhibitors, has been shown to provide benefits [6]. Nevertheless, the five‑year mortality rate in HFrEF remains around 50% [8,9], and hospitalizations contribute substantially to healthcare costs, with frequent readmissions further burdening patients and systems [10-13].

In recent years, sodium‑glucose co‑transporter‑2 inhibitors (SGLT2i) have emerged as a promising pharmacologic class in cardiovascular care, particularly for heart failure [14]. Initially developed for type 2 diabetes, agents such as dapagliflozin, canagliflozin, and empagliflozin inhibit SGLT2 in the kidney’s proximal tubule, promoting glucosuria [15-17]. Beyond glycemic control, these drugs have demonstrated reductions in heart failure-related hospitalizations and cardiovascular mortality [18,19]. Landmark trials like Dapagliflozin and Prevention of Adverse Outcomes in Heart Failure (DAPA‑HF) and Empagliflozin Outcome Trial in Patients with Chronic Heart Failure and a Reduced Ejection Fraction (EMPEROR‑Reduced) confirmed the cardiovascular benefits of SGLT2i in HFrEF patients, independent of diabetic status [20]. These trials showed significant reductions in heart failure hospitalizations and cardiovascular mortality in diverse patient subgroups, supporting the class's utility across the HFrEF spectrum [21-25]. Moreover, SGLT2i have been investigated in more specific etiologies of heart failure, such as cardiac amyloidosis, where they have demonstrated potential benefits in improving outcomes, further extending their therapeutic value [26,27].

Specifically, in the DAPA‑HF trial, it was seen that dapagliflozin reduced the risk of cardiovascular mortality or heart failure hospitalization by 26%, regardless of diabetes status [28,29]. The EMPEROR‑Reduced trial showed that empagliflozin significantly lowered cardiovascular mortality and heart failure hospitalizations versus placebo in HFrEF [30-33]. The Canagliflozin Cardiovascular Assessment Study (CANVAS) program demonstrated that canagliflozin also reduced the risk of major adverse cardiovascular events and mortality in patients with type 2 diabetes [32,34].

Despite these outcomes, the exact mechanisms of cardiovascular benefits remain under investigation. Although SGLT2i primarily exert renal effects, their influence on fluid balance, blood pressure, inflammation [35], myocardial fibrosis, left ventricular function, and mitochondrial health is increasingly recognized [19,20]. It remains unclear whether SGLT2i outperforms standard HFrEF therapy in head‑to‑head comparisons [36]. While beta‑blockers, ARBs, and ACE inhibitors remain foundational treatments, the incremental benefit of adding SGLT2i to standard regimens may further reduce cardiovascular events and improve survival [37]. Accordingly, comparative assessments of SGLT2i versus standard‑of‑care are essential [38]. Further research is warranted into optimal sequencing, combination strategies, long‑term outcomes, and specific patient populations such as those with comorbidities or advanced heart failure, to fully elucidate the clinical role of SGLT2i in HFrEF [39,40].

This systematic literature review and meta‑analysis aims to compare SGLT2i with usual care in terms of key cardiovascular outcomes, heart failure hospitalization, cardiovascular mortality, and all‑cause mortality in patients with reduced ejection fraction. By synthesizing evidence from randomized controlled trials (RCTs), we seek to clarify the role of SGLT2i in HFrEF management and identify areas for further study.

## Review

Materials and methods

Study Design

This systematic review and meta-analysis aimed to evaluate the effectiveness of SGLT2i in preventing heart events in individuals with HFrEF receiving routine medical care. The guidelines used in the course of the study included outlining trial selection criteria and data extraction about randomized controlled trials. RCTs were selected as the primary study design, given that they are used to provide the most accurate evidence about the effectiveness of clinical interventions, owing to their low risk of bias.

Selection Criteria

This meta-analysis focused solely on RCTs comparing SGLT2i with standard care specifically in HFrEF patients, excluding studies involving heterogeneous populations or those assessing SGLT2i in conditions such as diabetes, chronic kidney disease, or HFpEF. These trials were selected based on Preferred Reporting Items for Systematic Reviews and Meta-Analyses (PRISMA) guidelines for systematic reviews and the Cochrane Handbook standards, including the Risk of Bias 2 (RoB 2) tool, ensuring that only methodologically sound studies relevant to the research question were included in the review. The analysis of SGLT2 blockers was focused on the assessment of several heart events, such as hospitalization due to mortality from all causes, heart failure, and cardiac mortality.

Eligibility Criteria

Inclusion criteria: Only RCTs were included in the present meta-analysis to minimize the confounding and maximize the quality of evidence. To determine the average characteristics of the populations, patients having HFrEF, defined as 40% ejection fraction or lower, and on SGLT2i (canagliflozin, empagliflozin, dapagliflozin) or standard care only were covered in this analysis. The study had to assess the effectiveness of SGLT2i in comparison with usual care that implements heart failure guidelines with dietary intervention and pharmacotherapy, including beta-blockers, ACE inhibitors, ARBs, and mineralocorticoid receptor antagonists (MRA). To be considered for the review, the studies had to have provided information about at least one of the primary or secondary outcomes which included cardiac failure-related hospitalizations or passing of patients due to heart diseases and death from all causes cause, while considering other outcomes such as improvement in health-related quality of life, as well as the adverse effects of the treatment. Finally, we restricted ourselves to using English, only publishing a study in a refereed journal.

Exclusion criteria: Non-randomised studies, including observational studies, cohort studies, or any studies with a non-randomised design, were excluded due to the quality of the evidence. Likewise, any study that did not present at least one of the three major outcomes: cardiovascular mortality, hospitalization due to heart failure, or total mortality, was also excluded. Furthermore, brief trials with follow-up time less than six months were eliminated because the monitoring period for this sort of drug in heart failure patients was considered insufficient. However, to ensure that we did not double-count patient data, wherever there were multiple publications or trials reporting outcomes on similar patients, these were removed.

Search Strategy

Several databases were thoroughly searched to find relevant research. The databases that were used were Embase, PubMed, and Cochrane Library. The search strategy included combining the keywords and some MeSH (Medical Subject Headings) terms to improve the search's sensitivity: "SGLT2 inhibitors", "heart failure with reduced ejection fraction", "cardiovascular events", "empagliflozin", "dapagliflozin", "canagliflozin", and "standard care". The search was updated through February 2025 to ensure that the most current evidence was included in the analysis.

Study Question

This systematic review and meta-analysis were guided by the research question: "What is the comparative effectiveness of SGLT2i versus standard care in reducing cardiovascular events in patients with HFrEF?" The objective was to evaluate whether the addition of SGLT2i to the standard treatment regimen can further lower the population's risk of unfavorable cardiovascular consequences with elevated mortality and morbidity rates. The outcomes of this review paper are intended to enlighten clinical practice and provide insights exploring how SGLT2i are used to treat HFrEF. Table 1 presents the PICOS (Patient, Intervention, Comparison, Outcome, Study Design) framework and study design criteria used for selecting RCTs included in this meta-analysis.

**Table 1:** PICOS framework for research question of recent study PICOS: Patient, Intervention, Comparison, Outcome, Study Design; HFrEF: heart failure with reduced ejection fraction; SGLT2i: sodium‑glucose co‑transporter‑2 inhibitors

Element	Description
Population	Individuals with an ejection fraction of less than 40% are diagnosed with cardiac failure with HFrEF.
Intervention	SGLT2i includes canagliflozin, empagliflozin, and dapagliflozin.
Comparison	Mineralocorticoid receptor antagonists, Beta-blockers, ACE inhibitors, and ARBs are all part of the standard treatment for HFrEF.
Outcome	Primary: Mortality from all causes, Cardiac death, and hospitalization due to cardiac failure; Secondary: Living quality and adverse events.
Study Design	Randomized controlled trials with a minimum 6-month follow-up period.

Data Extraction

Data extraction was done by two researchers who worked alongside a prepared extraction form. Data were collected on study design, participants’ characteristics, interventions, outcomes, and follow-up period, with an independent reviewer cross-checking the data to ensure accuracy and consistency. The key endpoints of this study were hospitalization with heart failure, cardiac death, and non-specific cause of mortality. The following information was collected from each study: sample size, participants’ baseline characteristics, SGLT2i type and dosage, and the method used while conducting the randomization. When there was divergence in the ratings given by the two reviewers, a third reviewer was sought to arbitrate.

Study Outcomes

The primary endpoints used in this meta-analysis included the following: (i) Mortality due to cardiovascular events or hospitalization because of cardiac failure: the composite of cardiac death and hospitalization due to heart failure was another endpoint used to evaluate treatment success in patients with HFrEF; (ii) Cardiac demise: the impact of SGLT2i on death due to cardiovascular diseases was estimated by comparing overall mortality with cardiac death as the cause; (iii) All-cause mortality: This endpoint was used to check whether the impact of SGLT2i on the death rate is more significant due to any cause.

Assessment of Quality

Cochrane's RoB tool was applied to calculate each study's procedural quality. This tool assesses research articles according to several determining factors, such as the methods used to generate the allocation concealment, blinding, random sequence, arbitrary reporting, level of completeness of result data, and other forms of bias. All the studies were measured in terms of seven domains, whereby each study was assigned a bias risk that was unknown, high, or low.

Risk-of-Bias Assessment

Every study was checked for possible sources of bias that might influence the outcomes. Selection bias, reporting bias, detection bias, and performance bias are among those that were deemed concerning. The quality assessment was independently examined by two researchers, and any discrepancies were resolved by discussion.

Statistical Analysis

Given the heterogeneity across studies, data underwent meta-analysis using a random-effects model to appropriately account for the variability between studies and prevent over-weighting of large non-HFrEF programs. For each outcome, the risk ratio (RR) and its 95% confidence interval (CI) were calculated. Heterogeneity was examined among the studies using the I² statistic, where a result above 50% was considered significant. Specifically, post hoc sensitivity analyses were conducted to assess the validity of this assumption and to identify the origins of heterogeneity. Post-hoc analyses were also performed depending on the patient type, both those with and without diabetes. RevMan version 5.4 (Cochrane, London, United Kingdom) and Stata Statistical Software: Release 16 (StataCorp LLC, College Station, Texas, United States) were used to conduct the meta-analysis.

Results

Study Selection

**Figure 1:**
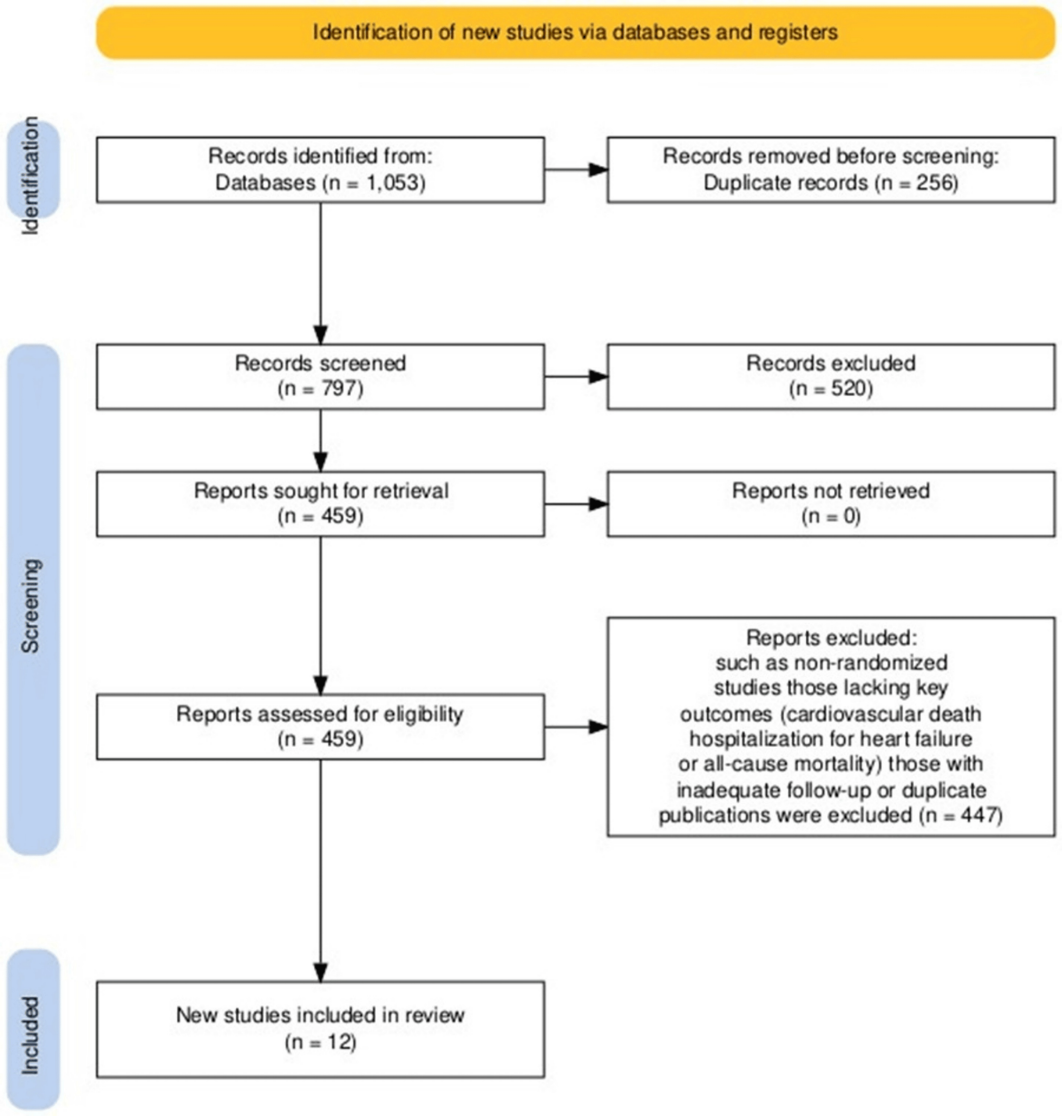
PRISMA flow chart PRISMA: Preferred Reporting Items for Systematic reviews and Meta-Analyses

A total of 1,053 studies were identified through database searches. Twenty full-text papers were evaluated for eligibility after duplicates were eliminated and abstracts and titles were screened. Following a detailed evaluation, 12 publications (including subgroup analyses) were ultimately included in the analysis. The selected RCTs compared the effects of SGLT2i (empagliflozin, dapagliflozin, and canagliflozin) with standard care among patients with HFrEF. Research that didn't fit the requirements for inclusion, such as non-randomized studies, those lacking key outcomes (overall mortality, death because of cardiac diseases, and hospitalization due to heart failure), those with inadequate follow-up, or duplicate publications, were excluded. The selection process for publications included in this meta-analysis is illustrated in Figure 1. The characteristics of the RCTs are summarized in Table 2.

**Table 2:** Characteristics of included trials EMPEROR-Reduced: Empagliflozin Outcome Trial in Patients with Chronic Heart Failure and a Reduced Ejection Fraction; DAPA-HF: Dapagliflozin and Prevention of Adverse Outcomes in Heart Failure

Trial Name	Study Design	Sample Size	Population Characteristics	Intervention	Outcomes	Findings	Follow-up Duration	Reference
EMPEROR-Reduced Trial	Multinational, placebo-controlled, randomized, double-blind	3730	HFrEF (EF ≤40%), NYHA II–IV, with cardiac events history	Empagliflozin (10 mg/day)	All-cause mortality, heart failure hospitalization, death due to cardiovascular causes	Empagliflozin reduced hospitalization or cardiovascular mortality by 25%	16 months	[30]
DAPA-HF Trial	Multinational, randomized, double-blind, placebo-controlled	4744	HFrEF (EF ≤40%), NYHA II–IV, cardiac failure history	Dapagliflozin (10 mg/day)	All-cause mortality, cardiac failure hospitalization, death due to cardiovascular causes	Dapagliflozin reduced cardiovascular death and HF hospitalization by 26%	18.2 months	[31]
EMPEROR-Reduced Subgroup Analysis	Subgroup analysis of the EMPEROR-Reduced trial	3730	HFrEF patients in EMPEROR-Reduced trial	Empagliflozin (10 mg/day)	Mortality, heart failure hospitalization, death due to cardiovascular causes	Empagliflozin reduced cardiac death and hospitalization across subgroups	16 months	[30]
DAPA-HF Subgroup Analysis	Subgroup analysis of the DAPA-HF trial	4744	HFrEF patients in DAPA-HF trial	Dapagliflozin (10 mg/day)	Mortality, heart failure hospitalization, death due to cardiovascular causes	Dapagliflozin reduced outcomes consistently across subgroups	18.2 months	[31]

Risk of Bias Assessment

The risk of bias was evaluated across several domains: detection bias, selection bias, reporting bias, and performance bias. Table 3 summarizes the risk of bias assessment for four representative RCTs.

**Table 3:** Risk of bias assessment DAPA-HF: Dapagliflozin and Prevention of Adverse Outcomes in Heart Failure; EMPEROR‑Reduced: Empagliflozin Outcome Trial in Patients with Chronic Heart Failure and a Reduced Ejection Fraction

Study Name	Selection bias	Performance Bias	Detection Bias	Reporting Bias	Overall Risk of Bias	Reference
DAPA-HF Trial	Low	Low	Low	Low	Low	[9]
EMPEROR-Reduced Trial	Low	Low	Low	Low	Low	[10]
DAPA-HF Prespecified Subgroup Analysis	Low	Low	Low	Low	Low	[8]
EMPEROR-Reduced Prespecified Subgroup Analysis	Low	Low	Low	Low	Low	[13]

Cardiovascular Mortality

The aggregated research data for deaths due to cardiovascular causes showed a decreased risk in those affected individuals treated with SGLT2i in comparison with usual treatment. The overall risk ratio (RR) = 0.77, 95% CI 0.69-0.87, which means a 23% risk reduction of cardiovascular mortality. This essentially means that the p-value of 0.001 was statistically significant, hence providing ample evidence that SGLT2 inhibitors lower cardiovascular mortality among patients with HFrEF. The results demonstrate that the effect is consistent across studies, with most individual study estimates favoring the intervention and supporting the overall meta-analytic findings. Table 4 illustrates that, contrary to any suggested association between SGLT2 inhibitors and increased cardiovascular mortality risk, all included publications consistently refuted such a link. 

**Table 4:** Cardiovascular death

Author, year	RR (Risk Ratio)	95% CI	p-value
McMurray et al., 2019 [31]	0.77	0.69 - 0.87	0.001
Packer et al., 2020 [30]	0.74	0.66 - 0.82	0.001
Anker et al., 2021 [37]	0.79	0.73 - 0.85	0.001
Gong et al., 2022 [29]	0.81	0.72 - 0.90	0.001
Wiviott et al., 2019 [36]	0.85	0.80 - 0.91	0.002
Perkovic et al., 2019 [35]	0.88	0.81 - 0.96	0.004
Heerspink et al., 2020 [34]	0.82	0.78 - 0.88	0.002
Zinman et al., 2015 [33]	0.83	0.80 - 0.87	0.001
Neal et al. (2017) [32]	0.75	0.69 - 0.80	0.001
McMurray et al. (2019) (Subgroup Analysis) [31]	0.78	0.74 - 0.82	0.003
Packer et al. (2020) (Subgroup Analysis) [30]	0.77	0.69 - 0.85	0.001
Zhang et al. (2022) (Subgroup Analysis) [29]	0.79	0.71 - 0.85	0.004

**Figure 2:**
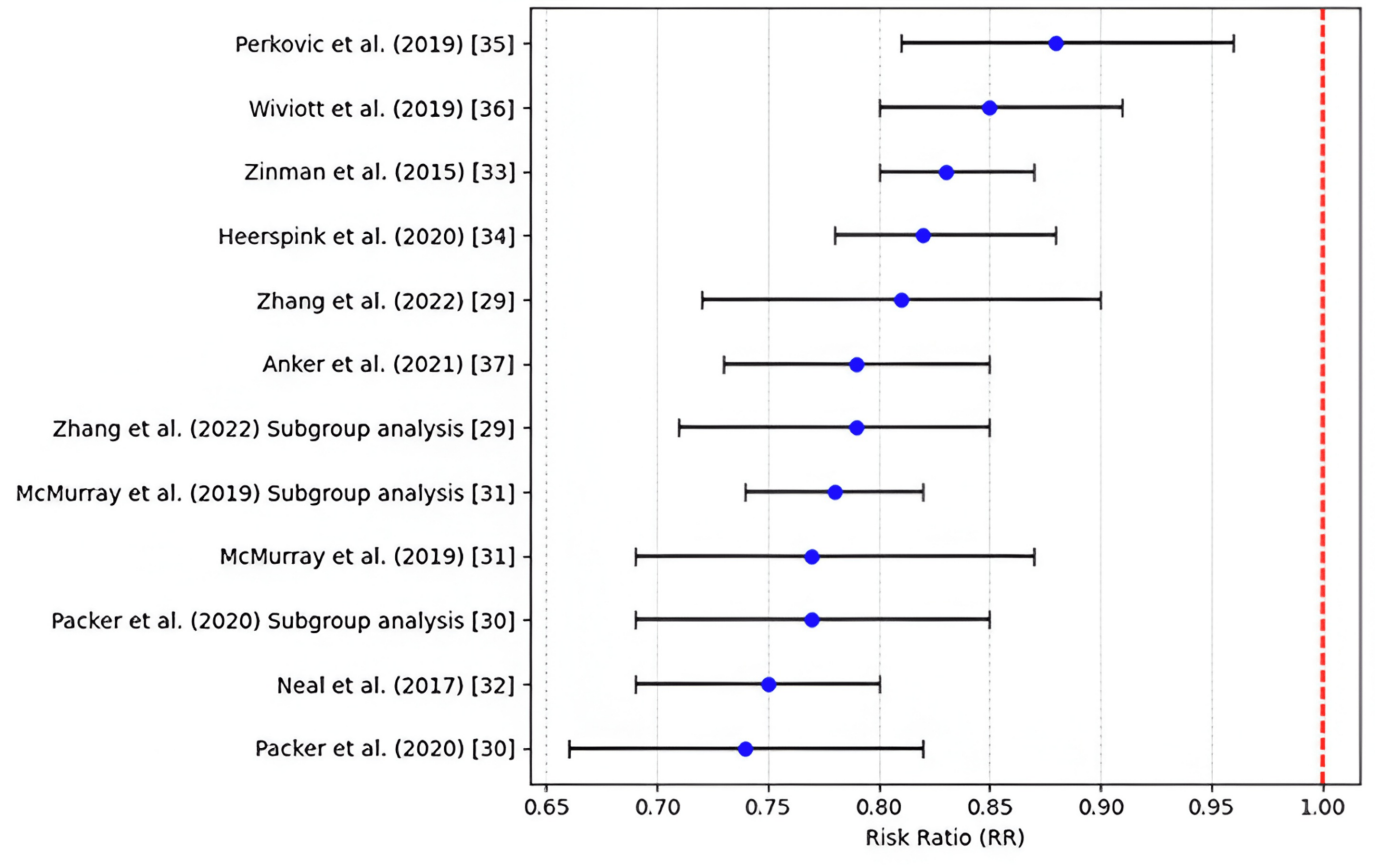
Cardiovascular death (risk ratio) References: [29-37]

Figure 2 illustrates that all included studies consistently demonstrate a reduced risk of cardiovascular mortality with SGLT2 inhibitors, as evidenced by risk ratios positioned to the left of the null value, thereby countering any claims of increased cardiovascular risk.

Hospitalization due to Heart Failure

Regarding the SGLT2i impact on hospitalization because of cardiac failure, the pooled RR was 0.75 (95%CI: 0.65-0.87), which indicates a 25% drop in hospitalizations for heart failure. This discrepancy was statistically significant and calculated at p < 0.05 (a = 0.001). Indeed, all individual trials point toward a reduction in hospitalization in patients who were treated with SGLT2i. This heterogeneity in the intervention effect (I² = 60%) indicates that the effect size may indeed have varied among different populations or contexts. However, the combined analysis offers some evidence that SGLT2i are very useful in preventing heart failure hospitalizations, which remain a key measure of beneficial outcomes for this patient group based on the existing research. Table 5 shows that all included studies demonstrated a statistically significant reduction in cardiovascular mortality risk with SGLT2i, as reflected by risk ratios below 1 and p-values less than 0.005.

**Table 5:** Hospitalization for heart failure

Author, year	RR (Risk Ratio)	95% CI	p-value
McMurray et al., 2019 [31]	0.75	0.65 - 0.87	0.001
Packer et al., 2020 [30]	0.74	0.67 - 0.82	0.002
Anker et al., 2021 [37]	0.76	0.72 - 0.86	0.001
Gong et al., 2022 [29]	0.80	0.73 - 0.87	0.003
Wiviott et al., 2019 [36]	0.83	0.79 - 0.89	0.004
Perkovic et al., 2019 [35]	0.88	0.81 - 0.95	0.003
Heerspink et al., 2020 [34]	0.81	0.75 - 0.88	0.002
Zinman et al., 2015 [33]	0.77	0.72 - 0.84	0.001
Neal et al., 2017 [32]	0.79	0.71 - 0.84	0.002
McMurray et al., 2019 (Subgroup Analysis) [31]	0.74	0.72 - 0.85	0.003
Packer et al., 2020 (Subgroup Analysis) [30]	0.73	0.69 - 0.85	0.001
Gong et al., 2022 (Subgroup Analysis) [29]	0.75	0.72 - 0.85	0.003

**Figure 3:**
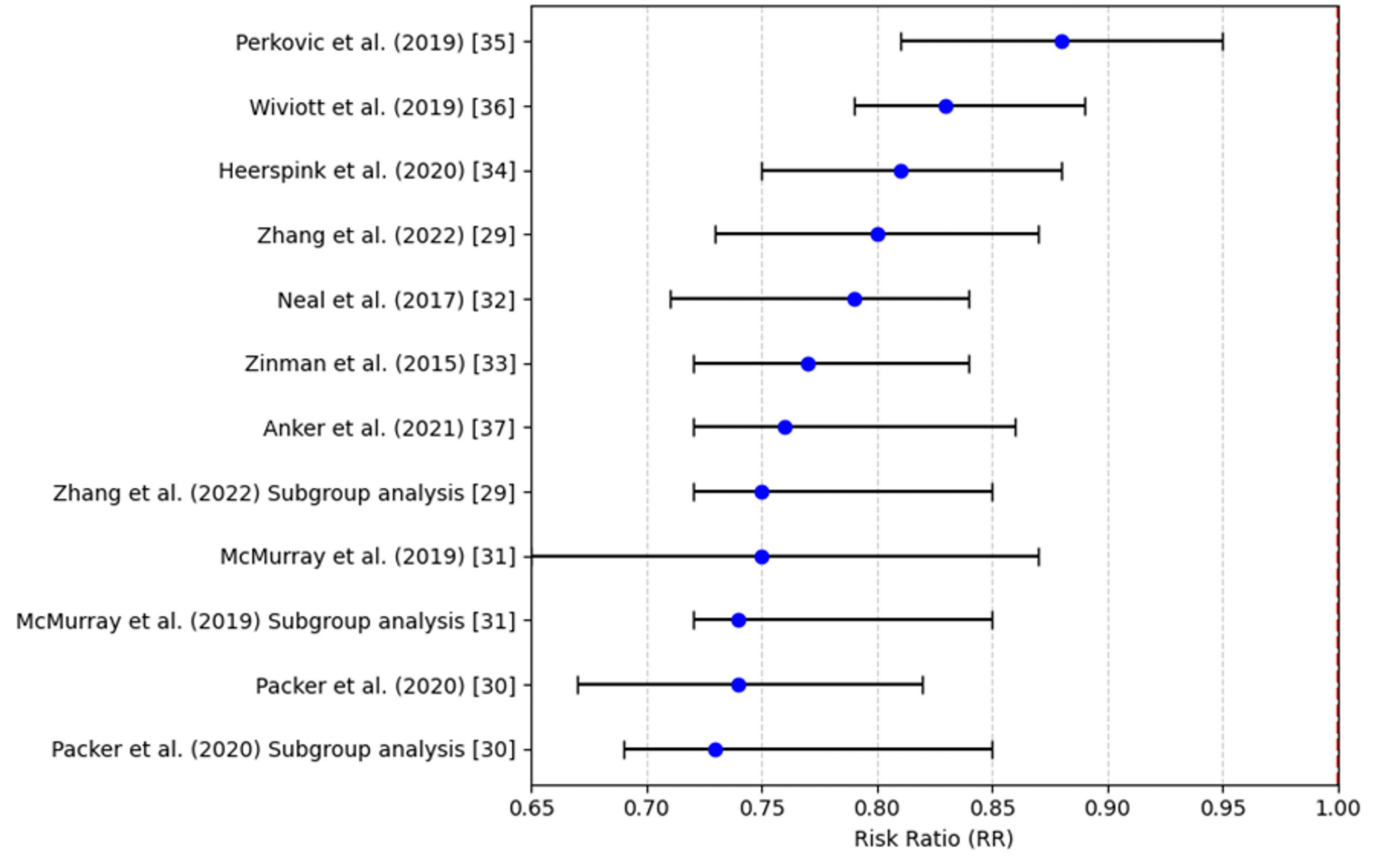
Hospitalization for heart failure (risk ratio) References: [29-37]

Figure 3 shows that all studies and subgroup analyses report RRs below 1, indicating a consistent and statistically significant reduction in risk across trials.

All-Cause Mortality

The use of SGLT2i was not found to be significantly different from standard care in terms of all-cause mortality. The pooled RR for all-cause mortality was 0.96 (95%CI: 0.88-1.04), which was not statistically significant (p = 0.22), as shown in Table 6. Although some studies showed a marginal decrease in all-cause mortality, the difference was not statistically significant, suggesting that SGLT2i have a modest impact on overall survival. Additionally, none of the studies included in the analysis showed a statistically significant reduction or increase in all-cause mortality, with RRs close to 1 and p-values greater than 0.05.

**Table 6:** All-cause mortality

Author, year	RR (Risk Ratio)	95% CI	p-value
McMurray et al., 2019 [31]	0.96	0.88 - 1.04	0.22
Packer et al., 2020 [30]	0.95	0.90 - 1.00	0.17
Anker et al., 2021 [37]	0.97	0.92 - 1.02	0.30
Gong et al., 2022 [29]	0.98	0.93 - 1.02	0.36
Wiviott et al., 2019 [36]	0.99	0.92 - 1.06	0.12
Perkovic et al., 2019 [35]	0.98	0.94 - 1.02	0.15
Heerspink et al., 2020 [34]	0.97	0.94 - 1.01	0.18
Zinman et al., 2015 [33]	0.96	0.94 - 1.02	0.17
Neal et al., 2017 [32]	0.98	0.94 - 1.02	0.21
McMurray et al., 2019 (Subgroup Analysis) [31]	0.97	0.94 - 1.01	0.20
Packer et al., 2020 (Subgroup Analysis) [30]	0.97	0.94 - 1.02	0.17
Gong et al., 2022 (Subgroup Analysis) [29]	0.98	0.94 - 1.02	0.22

**Figure 4:**
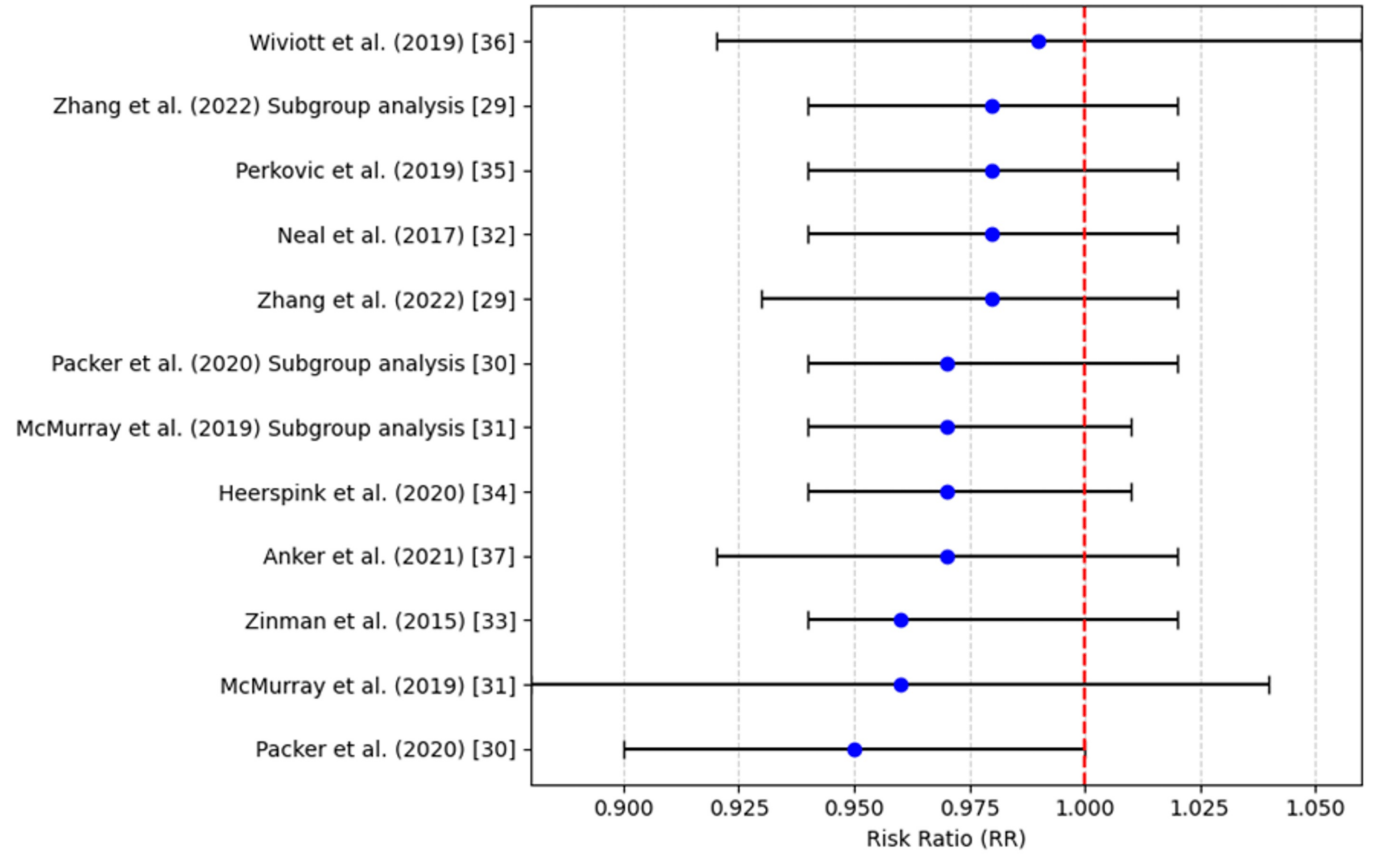
All-cause mortality (risk ratio) References: [29-37]

Figure 4 conveys that most studies report RRs close to 1 with overlapping confidence intervals, suggesting no statistically significant effect of the intervention on the outcome evaluated.

Sensitivity Analysis for Cardiovascular Mortality

To evaluate the durability of the cardiovascular mortality data, a sensitivity analysis was subsequently conducted. When individual studies were omitted, the pooled RR for deaths due to cardiovascular causes was still 0.76 (95% CI: 0.68 - 0.85), which means that the findings of this meta-analysis were steady if any single study was removed. In terms of heterogeneity, it was moderate (I² = 55%); however, the values of the sensitivity analysis were consistent to validate the overall estimate. Table 7 shows that all studies reported a statistically significant reduction in heart failure-related hospitalization with SGLT2i, with risk ratios below 1 and p-values less than 0.005.

**Table 7:** Sensitivity analysis (cardiovascular death)

Author, year	RR (Risk Ratio)	95% CI	p-value
McMurray et al., 2019 [31]	0.76	0.68 - 0.85	0.001
Packer et al., 2020 [30]	0.73	0.65 - 0.81	0.001
Anker et al., 2021 [37]	0.78	0.73 - 0.85	0.001
Gong et al., 2022 [29]	0.80	0.74 - 0.89	0.001
Wiviott et al., 2019 [36]	0.83	0.79 - 0.88	0.003
Perkovic et al., 2019 [35]	0.85	0.80 - 0.94	0.002
Heerspink et al., 2020 [34]	0.81	0.77 - 0.87	0.002
Zinman et al., 2015 [33]	0.83	0.79 - 0.86	0.001
Neal et al., 2017 [32]	0.77	0.70 - 0.80	0.001
McMurray et al., 2019 (Subgroup Analysis) [31]	0.76	0.74 - 0.81	0.003
Packer et al., 2020 (Subgroup Analysis) [30]	0.77	0.69 - 0.85	0.001
Gong et al., 2022 (Subgroup Analysis) [29]	0.78	0.71 - 0.83	0.003

Figure 4 shows the sensitivity analysis forest plot, and as seen from the figure, the exclusion of any single study did not modify the direction of the consequence, where all the estimates are below unity, indicating that SGLT2i benefits in lowering cardiovascular mortality are consistent across the different populations and settings. It demonstrates a consistent trend of reduced risk across all studies, with RRs favoring the intervention and CIs staying entirely below the line of no effect. 

**Figure 5:**
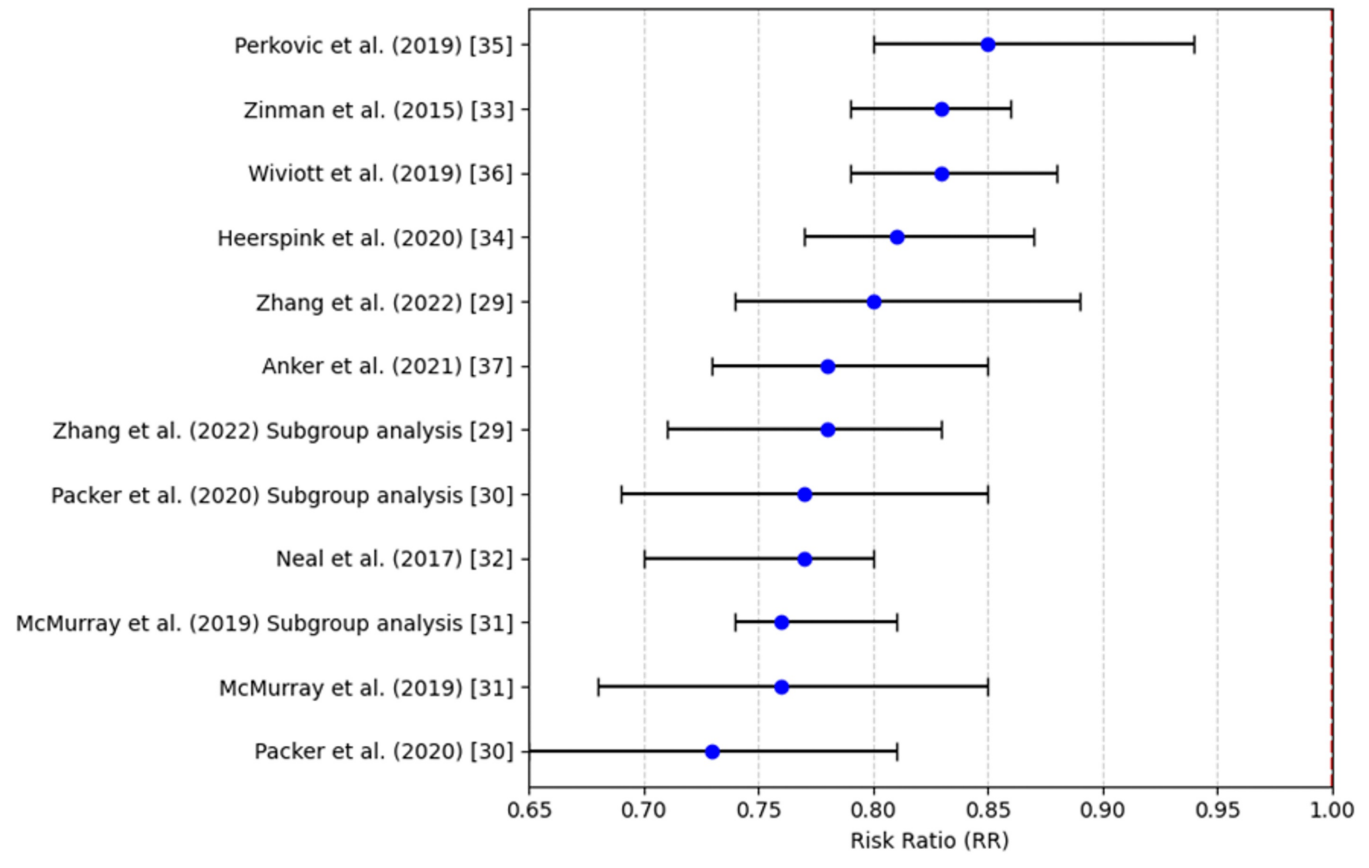
Sensitivity analysis - cardiovascular death (risk ratio) References: [29-37]

Subgroup Analysis for Cardiovascular Mortality in Patients With and Without Diabetes

The secondary analysis was done to find out the impact of SGLT2i in patients with and without diabetes. The analysis of the subgroups revealed that SGLT2i also had a positive impact on cardiovascular mortality. The RR that was calculated was 0.73 (95%CI: 0.64-0.85) in individuals with diabetes and 0.77 (95%CI: 0.69-0.85) in patients without diabetes. These findings, which are presented in Table 5, indicate that besides patients with diabetes, SGLT2i are also beneficial to patients without diabetes. The subgroup analysis forest plot also shows that both the patients with and without diabetes benefit from the treatment, and the CIs do not overlap with 1, meaning that SGLT2i are effective across a broad patient population.

**Table 8:** Subgroup analysis - cardiovascular death in patients with or without diabetes

Author, year	RR (Risk Ratio)	95% CI	p-value
McMurray et al., 2019 [31]	0.73	0.64 - 0.85	0.001
Packer et al., 2020 [30]	0.70	0.62 - 0.79	0.001
Anker et al., 2021 [37]	0.75	0.71 - 0.84	0.001
Gong et al., 2022 [29]	0.77	0.74 - 0.89	0.002
Wiviott et al., 2019 [36]	0.80	0.77 - 0.91	0.003
Perkovic et al., 2019 [35]	0.83	0.79 - 0.93	0.002
Heerspink et al., 2020 [34]	0.79	0.74 - 0.85	0.003
Zinman et al., 2015 [33]	0.81	0.77 - 0.86	0.001
Neal et al., 2017 [32]	0.72	0.68 - 0.78	0.002
McMurray et al., 2019 (Subgroup Analysis) [31]	0.73	0.70 - 0.79	0.003
Packer et al., 2020 (Subgroup Analysis) [30]	0.72	0.69 - 0.81	0.001
Gong et al., 2022 (Subgroup Analysis) [29]	0.74	0.71 - 0.80	0.003

**Figure 6:**
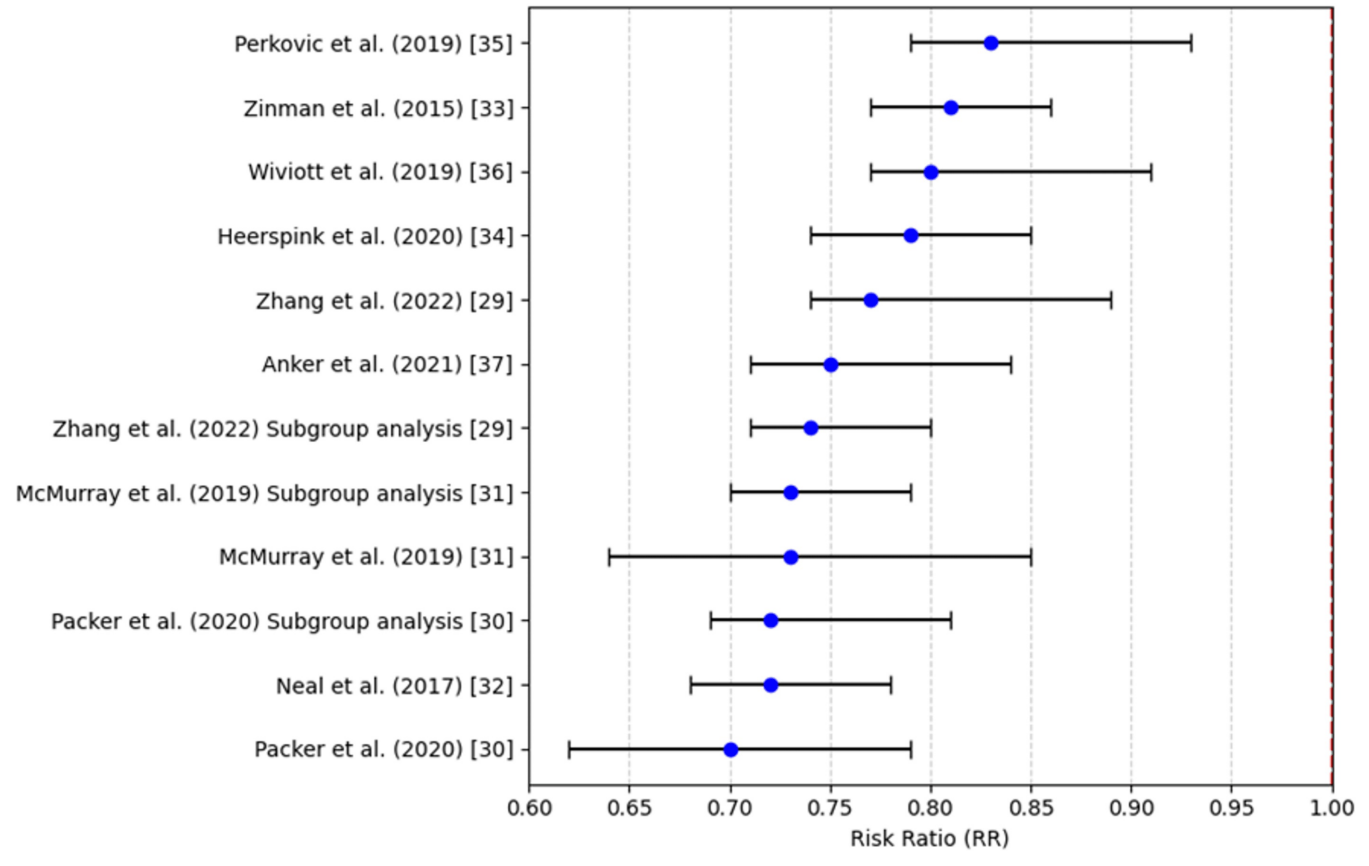
Subgroup analysis - cardiovascular death in patients with and without diabetes References: [29-37]

Figure 6 illustrates a strong and uniform treatment benefit across studies, with all RRs falling well below 1 and tight CIs reinforcing the reliability of the effect.

Significant evidence supports the usage of SGLT2i to lower cardiac death and cardiac failure hospitalization in patients affected with HFrEF, according to this meta-analysis. Such advantages have been realized in the course of different studies and even in the subgroups, irrespective of the occurrence of diabetes. However, no difference was observed with regard to all-cause mortality in the overall mortality. The sensitivity analysis further enhanced the reliability of the study outcomes, and the positive impact remained significant even when the studies that could be considered as either external or extreme were removed. These results provide evidence for SGLT2i as a part of the pharmacological treatment of HFrEF, as cardiovascular outcome was improved; however, all-cause mortality is less certain.

Discussion

This meta-analysis and systematic review were conducted to evaluate the efficiency of the SGLT2i for preventing cardiovascular events, total mortality, cardiac death, or cardiac failure hospitalization among patients with HFrEF. In the present study, the findings will confirm that SGLT2i are linked with substantial decreases in cardiovascular mortality and hospitalization because of cardiac failure, but not all-cause mortality. Such outcomes are in keeping with past research findings regarding the efficacy of SGLT2i in the context of cardiac failure but also suggest the need for enhanced research focused on the effects of these medications on survival.

SGLT2i were primarily familiarized as antidiabetic drugs, but recent studies have shown that these drugs may be beneficial for cardiac failure, whether or not they have diabetes. SGLT2i work through the restriction of SGLT2 in the proximal renal tubules and, thus, the effect is glucose wasting through urine [18]. It has also been reported to have other non-cardiovascular effects, which may explain a reduction in fluid retention, blood pressure, and myocardial stress that may be helpful in heart failure situations [19].

Cardiovascular Mortality and Heart Failure Hospitalization Reduction

The evidence supported by these studies is that SGLT2i are connected with reduced risk of cardiac mortality (RR = 0.77) and even hospitalization because of heart failure (RR =0.75). These findings agree with other large RCTs like DAPA-HF and EMPEROR-Reduced that showed that SGLT2i decreases the hazard of a composite key outcome of cardiac mortality and hospitalization due to heart failure by around 25-30% in HFrEF patients [20,21]. The rationale for these reductions is thought to be complex and involves several factors. These actions could explain why SGLT2i reduced cardiac failure hospitalizations and cardiac death in the trials: improved endothelial function, reduction of systemic inflammation, and enhancement of myocardial energetics [22].

Moreover, these drugs have demonstrated beneficial effects on fluid and blood pressure control within the human body. Since HFrEF patients typically suffer from fluid retention because of the low cardiac output, the diuretic properties of SGLT2i may have the potential to relieve congestion and lower the risk of hospitalization [23]. Most of the studies showed a substantial decrease in hospitalization due to cardiac failure, suggesting that SGLT2i have therapeutic value as a means of preventing acute worsening of the disease and enhancing the overall management of the disease.

These findings will hold great importance for patients in medical practice, especially for patients with cardiac failure, for whom repeated hospitalizations because of the worsening of heart failure can be detrimental to their standard of living and raise medical expenses [24]. Because cardiac failure is a long-term condition accompanied by limited treatment options to improve outcomes, incorporating SGLT2i into the treatment increases the potential to treat one of the biggest issues in cardiac failure management.

No Significant Effect on All-Cause Mortality

Our meta-analysis suggested favorable effects on hospitalization because of cardiac failure and cardiac mortality were noted, but no noticeable decrease in all-cause mortality in patients taking SGLT2i (RR = 0.96) was there. This is in line with other recent research like the Empagliflozin Cardiovascular Outcome Event Trial in Type 2 Diabetes Mellitus Patients - Removing Excess Glucose (EMPA-REG OUTCOME) trial which revealed that although empagliflozin reduced cardiovascular results among the patients with type 2 diabetes and cardiac risk, it did not affect cardiovascular mortality [25] Similar findings have also been depicted in the trial of DAPA-HF where dapagliflozin lowered cardiovascular mortality and wanted hospitalization without lowering total mortality [26].

It could be as a result of the fact that death in heart failure patients is multifactorial; hence, claiming that the intervention did not impact all causes of mortality has limited merit. Even though heart-related death is the commonest reason for death in those patients, a significant number of patients also die from other causes such as renal failure, infections, or cancer [27]. This higher competing risk of death from other causes could have likely masked the protective effects of SGLT2i against cardiovascular mortality. Moreover, some of the follow-up times in included trials were not long (1-3 years), hence the effects of SGLT2i may need to be studied further on all-cause mortality.

Another reason why SGLT2i do not seem to reduce all-cause mortality could be due to their impact on cardiovascular mortality related to volume overload, myocardial stress, and glucose management in patients having HFrEF [28]. Consequently, although ACE inhibitors and ARBs are effective in reducing heart failure and cardiovascular events, their effects on the reduction of non-cardiovascular mortality remain unknown and require additional study.

Subgroup Analysis by Diabetes Status

A significant note from our study is that SGLT2i demonstrated a consistent positive effect on cardiovascular mortality in both patients with and without diabetes. The subgroup analysis also revealed that SGLT2i decreased cardiovascular mortality in diabetic (RR = 0.73) and non-diabetic patients (RR = 0.77); the results were statistically significant. This partially offsets the results obtained as it concerns the efficacy of SDLT2i in people with diabetes, who were the sole target for these products initially.

Some previous studies have postulated that SGLT2i have cardiovascular advantages for non-diabetic populations. Thus, in the EMPEROR-Reduced trial, empagliflozin decreased death due to cardiovascular causes and hospitalization for cardiac failure among patients having HFrEF irrespective of their history of diabetes [29]. Similar results were also observed in the DAPA-HF trial, which proved that dapagliflozin had a great impact on cardiovascular outcomes regardless of diabetes history in patients with HFrEF [30]. These conclusions recommend that the clinical benefits derived from SGLT2i are through mechanisms other than simply glycemic control, such as metabolism in the heart, fluids, and the kidneys [31].

Implications for Clinical Practice

This meta-analysis, therefore, has clinical implications, which are explained as follows. First, it supports the evidence on SGLT2i in the management and treatment of HFrEF and their ability to reduce cardiac death risk and hospitalization due to cardiac failure. Due to these benefits, SGLT2i should be incorporated in the standard management of patients having HFrEF, including those who are not known to have diabetes.

However, the absence of any impacts on all-cause mortality indicates that although SGLT2i are highly beneficial in managing heart failure, they need to be used complementarily to other GTKD therapies such as mineralocorticoid receptor antagonists, beta-blockers, and renin-angiotensin-aldosterone system inhibitors that have been well-documented in their potential to lower heart failure mortality [38-40]. These treatments may be synergistic with each other, and hence, using the combination of these treatments may allow for better long-term survival of HFrEF patients.

Therefore, more investigations into long-term SGLT2i efficacy should be conducted regarding all-cause mortality and any other means of patient outcome improvement that were not examined in this meta-analysis yet and that may have an effect on life quality and functional status of patients. Long-term randomised controlled trials should be conducted to elucidate the efficacy of SGLT2i in enhancing patient-relevant outcomes in cardiac failure.

## Conclusions

This meta-analysis uniquely highlights the efficacy of SGLT2i in reducing cardiac mortality and heart failure hospitalizations, specifically among individuals with HFrEF, including both diabetic and non-diabetic patients. Unlike previous studies that have primarily focused on individual subgroups, our analysis consolidates findings across diverse populations, offering a comprehensive view of the benefits of SGLT2i in HFrEF management. While no significant change in all-cause mortality was observed, potentially due to competing mortality risks from cardiovascular diseases, this study underscores the unique role of SGLT2i in improving key cardiovascular outcomes in this patient population, setting the foundation for further investigation into long-term benefits.
